# Genetic engineering cellular vesicles expressing CD64 as checkpoint antibody carrier for cancer immunotherapy

**DOI:** 10.7150/thno.48868

**Published:** 2021-04-07

**Authors:** Liyan Li, Qianwei Miao, Fanqiang Meng, Baoqi Li, Tianyuan Xue, Tianliang Fang, Zhirang Zhang, Jinxie Zhang, Xinyu Ye, Yang Kang, Xingding Zhang, Qian Chen, Xin Liang, Hongbo Chen, Xudong Zhang

**Affiliations:** 1 Department of Pharmacology, Molecular Cancer Research Center, School of Medicine, Sun Yat-Sen University, Guangzhou/Shenzhen, China.; 2 Guangdong Provincial Key Laboratory of Medical Molecular Diagnostics, Key Laboratory of Stem Cell and Regenerative Tissue Engineering, School of Basic Medical Sciences, Guangdong Medical University, Dongguan 523808, China.; 3 School of Pharmaceutical Sciences (Shenzhen), Sun Yat-Sen University, Shenzhen, P.R. China.; 4 School of Life Sciences, Tsinghua University, Beijing 100084, P.R. China.; 5 The Seventh Affiliated Hospital, Sun Yat-sen University, Shenzhen, 518107, China.; 6 Institute of Functional Nano & Soft Materials (FUNSOM), Soochow University, Suzhou, 215325, P.R. China.; 7 Center for Experimental Medicine (CEM), University of Chinese Academy of Sciences-Shenzhen Hospital, Shenzhen 518000, P. R. China.

**Keywords:** Cancer immunotherapy, checkpoint antibody, CD64, Regulatory T cells, Nanovesicle.

## Abstract

Immune checkpoint blockade therapies, especially those targeting the programmed death-1 (PD-1)/programmed death-ligand 1 (PD-L1) have achieved impressive clinical responses in multiple types of cancers. To optimize the therapeutic effect of the checkpoint antibodies, many strategies including targeting delivery, controlled release, and cellular synthesis have been developed. However, within these strategies, antibodies were attached to drug carriers by chemical bonding, which may affect the steric configuration and function of the antibodies. Herein, we prepared cluster of differentiation 64 (CD64), a natural catcher of the fragment crystalline (Fc) of monomeric immunoglobulin G (IgG), and over-expressed it on the cell membrane nanovesicles (NVs) as PD-L1 antibody delivery vehicle (CD64-NVs-aPD-L1), which was employed to disrupt the PD-1/PD-L1 immunosuppressive signal axis for boosting T cell dependent tumor elimination. Meanwhile, chemical immunomodulatory drug cyclophosphamide (CP) was also encapsulated in the vesicle (CD64-NVs-aPD-L1-CP), to simultaneously restrain the regulatory T cells (Tregs) and invigorate Ki67^+^CD8^+^ T cells, then further enhance their anti-tumor ability.

**Methods**: The cell membrane NVs overexpressing CD64 were incubated with PD-L1 antibody and chemotherapeutic agent CP to prepare CD64-NVs-aPD-L1-CP.

**Results:** The CD64-NVs-aPD-L1-CP could simultaneously interrupt the immunosuppressive effect of PD-L1 and decrease the inhibition of Tregs, leading to tumor growth suppression and survival time extension.

Conclusion: CD64-NVs are charismatic carriers to achieve both checkpoint blockade and immunomodulatory drugs for combined cancer immunotherapy.

## Introduction

In recent years, immunotherapy has become a promising clinical treatment for cancer combining with surgery, chemotherapy and radiotherapy 1-6. Currently, immune checkpoint blockade therapy has achieved impressive clinical responses in multiple cancers, such as non-small cell lung cancer, melanoma, glioblastoma, bladder cancer and renal cell carcinoma 7-12. After years of developments, there are a couple of commercial checkpoint blockade antibodies, including Nivolumab (PD-1 antibodies), Avelumab (PD-L1 antibodies), and Ipilimumab (CTLA-4 antibodies) 13-17. Particularly, PD-1/PD-L1 signal axis is the most common and broad target for cancer treatment. PD-L1 has been found overexpressed in multiple types of cancers, and it can inhibit the activation and proliferation of the effector T cells 18-20. Therefore, preventing PD-1 from binding to PD-L1 by antibodies could promote antitumor immunotherapy 21-23. However, anti-PD-1/PD-L1 therapy is not always effective and powerful to cope with all kinds of tumors and only about 20-40% of the patients can benefit from it 24-26. Moreover, it should not to be neglected that excess antibodies can lead to autoimmune diseases 27. Hence, how to improve the therapeutic effective and specific of the checkpoint antibody becomes an urgent issue. The drug delivery strategy has focused on reducing side effects and improves the therapeutic effect. To achieve these benefits, PD-1 antibodies were conjugating the platelets or platelet decorated hematopoietic stem cells to enhance the therapeutic effect of by with PD-1 antibodies 28. Moreover, it can promote PD-L1 antibodies by wound targeting delivery to the tumor sites with postoperative resection to inhibit tumor relapse and metastasis 29, 30.

Cell membrane vesicles derived from erythrocytes, NK cells, tumor cells, platelets and stem cells, are considered as promising drug carriers to target tumor cells 31, 32. Their good performance on biocompatibility, fluidity and circulatory stability, as well as the ability to cross biological barriers makes the exosome-like cell membrane NVs as attractive drug vehicles 33-36. Particularly, genetically engineered cellular membrane can display naturally full-length antibodies, receptors or ligands, which can maintain their natural three-dimensional structures and functions 37, 38. Meanwhile, they inherit the components and functions of original cell membrane, enabling them act as bioactive drug carriers to combine chemotherapy and immunotherapy for targeted anticancer treatment 37-39. The cell membrane conjugating PD-L1 antibodies precede free PD-L1 antibodies in the ability of tumor cells combination and the release control, since only a portion of free PD-L1 can successfully bind to the tumor cells and their aggregation concentration is usually affected by various factors 28. Moreover, the cell membrane NVs can express antibodies inherently instead of binding chemically, such as thiol-maleimide chemistry, which endows them with better orientation, powerful reactivity, and protection from destructing the active sites 28, 33, 38.

**Scheme 1:**
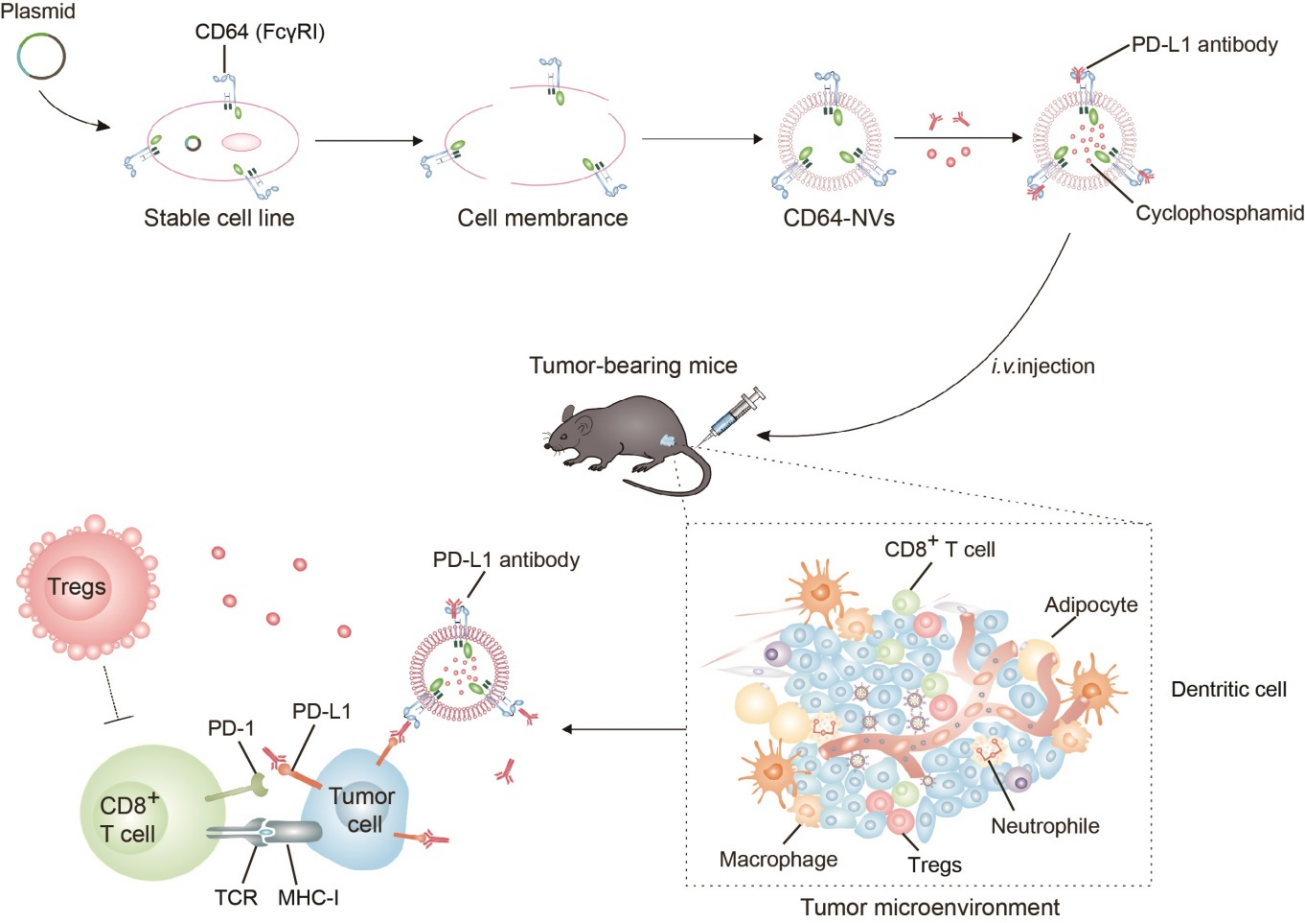
Schematic of preparation of CD64-NVs-aPD-L1-CP and immune boosting mechanism of NVs. CD8^+^ T cells were activated by CD64-NVs-aPD-L1-CP through binding to PD-L1 on tumor cells and inhibiting Tregs activity.

CD64, also referred to FcγRI, which is naturally expressed on the macrophages, monocytes, and dendritic cells, has high affinity to bind the Fc of monomeric IgG in nanomolecular range 40. Once CD64 on macrophages binds to the antibodies, it can regulate immune response and disease development by affecting phagocytosis 41-43. In addition, Tregs inhibit effector T cells and the activation and proliferation of CD8^+^ T cell by consuming interleukin-2 (IL-2) and releasing perforin and granzyme 44-46. Herein, we prepared CD64 presenting cellular NVs, which can correctly combine with the Fc of PD-L1 antibody. CD64-NVs, conjugating with PD-L1 antibodies, can achieve tumor targeting delivery. PD-L1 antibodies execute PD-L1 blockade, thereby promoting CD8^+^ T cells to attack cancer cells. CP is a broad-spectrum anticancer drug; low dosage of CP could inhibit Tregs to reinvigorate CD8^+^ lymphocyte cells, directly enhancing anti-tumor ability of immune system 47-49. The chemotherapeutic agent CP was encapsuled into CD64-NVs-aPD-L1 to facilitate CD8^+^ T cell forcefully and kill tumor cells through overcoming the obstacles of PD-1/PD-L1 axis and reverting Tregs suppression (Scheme 1).

## Materials and methods

### Reagents

Murine CD64 antibody for western blot and HPR labeled secondary antibody were from R&D Systems and KPL, respectively. GAPDH antibody was from Abmart. CD8 and CD4 antibodies for immunohistochemical staining and Alex Fluor 647 and Alex Fluor 488 labeled secondary antibody were purchased from Abcam. CD25, Ki67, Foxp3 antibodies for flow cytometry analysis and PD-L1 antibody for western blot were from Biolegend Inc. FITC-CD3 antibody was from Ebioscience. PE-CD4 and APC-CD8 antibodies were purchased from Life. Cyclophosphamide and hygromycin were from Sigma-Aldrich. Wheat Germ Agglutinin (WGA) Alexa Fluor 488 and 594 dyes were from Thermo Fisher.

### Cell Culture

HEK 293T cells were cultured in Dulbecco's modified Eagle's medium (DMEM) containing 10% fetal bovine serum (FBS) and 1% penicillin/streptomycin.

### Plasmid and Stable Cell Line

The pCMV6 mammalian expression vector encoding murine CD64 was fused with EGFP tag on the C terminal (Sino Biological Inc). The CD64 plasmids were transiently transfected into HEK 293T cells with lipofectamine 2000 (Invitrogen) and the expression of CD64 was identified by confocal and western blot. To establish stable CD64 expressing HEK 293T cell line, different concentrations of hygromycin were further used to select stable cell line for seven days. Established EGFP-CD64 HEK 293T cells were maintained in DMEM complementary with 10% FBS and 200 µg/mL hygromycin.

### Preparation of CD64-cell Membrane Nanovesicle

The HEK 293T cells stably expressing EGFP-CD64 were cultured in DMEM with 10% FBS and 200 μg/mL hygromycin and harvested with trypsin. After being washed with cold PBS for two times and centrifuged at 1000 rpm, the cells were suspended with homogenization medium (HM) (pH 7.4) including 20 mM Hepes-NaOH, 1 mM EDTA, 0.25 M sucrose, and protease inhibitor. Afterwards, the cells were ruptured on ice at least 150 times using a dounce homogenizer. Then, the liquid in the homogenizer was collected and centrifuged at 1000g for 5 min and followed on centrifuging the supernatant at 3000g for 5 min. And then, the supernatant was centrifuged at 20000 rpm for 30 min. Next, the precipitation with HM solution was resuspended and extruded through the 0.8 μm and 0.22 μm filters for at least seven times, respectively.

### Flow Cytometry

The cells stably expressing EGFP-CD64 were digested, centrifuged, and resuspended with 1 mL PBS. Then the cells were filtered by cell sieve to make them become unicellular. Flow detection was performed directly for further analysis to determine the positive rate of GFPSpark-CD64 expression.

### Western blot

HEK 293T control cells and HEK 293T cells stably expressing CD64 were lysed with 1x protein loading buffer and boiled at 100 ℃ for 15 min. And then, cell lysates and purified cell vesicles proteins were separated using 12% SDS-PAGE and transferred to PVDF membranes. The membranes were blocked with 5% milk for 1 h at room temperature and washed with PBS for 5 min. Then the membranes were incubated with CD64 antibodies overnight at 4 ℃ and washed with PBS for 5 min. The membranes were incubated with the secondary antibodies for 1 h at room temperature and washed with PBS for 5 min. Immunoblotting was analyzed by enhanced chemiluminescence detection (Thermo Scientific).

### EGFP-CD64-aPD-L1 Binding Assay

EGFP-CD64-NVs were incubated with PD-L1 antibodies for 4 h and then PD-L1 antibodies were stained with Alex Fluor 647. The sample was dropped onto the slide, sealed with adhesive, and observed directly under the confocal laser microscope. HEK 293T cells stably expressing CD64 were inoculated in glass slides. After cell adherence, the cells were incubated with PD-L1 antibodies for 4 h. Alexa Fluor 647 conjugate and DAPI were added to label PD-L1 antibodies and nucleus for 10 min, respectively. After being washed with PBS three times for 5 min each, the slides were directly observed under the confocal laser microscope.

### CD64-NVs loaded aPD-L1 and/or CP

After the preparation of CD64-NVs, the vesicles were quantified using BCA assay. 1 mg CD64-NVs were incubated with 100 μg aPD-L1 and/or 500 μg CP at 37 ℃ for 4 h. After incubation, the NVs were washed in PBS by centrifugation at 12,000 rpm for three times. The precipitate was resuspended with 150 μL PBS and injected into a mouse.

### Released of aPD-L1 and CP

To explore the release of PD-L1 antibodies and CP *in vivo*, we measured the release amount through simulation environment. After loading of PD-L1 antibodies and CP, we divided it evenly into several tubes, with three tubes at each point in time. At 37 ℃ shaker condition with the 150 rpm speed, we collect samples at different time points through centrifuging to take the supernatant at the condition of 14800 rpm, 4 ℃, 30 min. Western blot analysis was used to determine the release of PD-L1 antibodies from CD64-NVs at different time points (0.5 h, 1 h, 2 h, 4 h, 8 h and 12 h). The release of CP from CD64-NVs was analyzed in PBS at different time points at 37 °C (1 h, 4 h, 8 h, 12 h, 24 h and 48 h). The amount of CP released was quantified by using a UV-vis spectrophotometer at 205 nm.

### Circulation

After CD64-NVS being labeled with NHS-Cy5.5 overnight at 4 ℃ and washed in PBS for three times, the labeled CD64-NVs were incubated with aPD-L1 at 37 ℃ for 2 h. After incubation, the NVs were washed in PBS for three times. The CD64-NVs and CD64-NVs-aPD-L1 were injected into C57BL/6J mice through tail-vein, respectively. The orbital blood was collected at different time points (2 min, 20 min, 40 min, 1 h, 2 h, 4 h, 6 h, 16 h and 24 h) after injection and the fluorescence signal was measured.

### Biodistribution

CD64-NVs-aPD-L1 and CD64-NVs produced from CD64-HEK 293T cell line were labeled by NHS-Cy5.5 in PBS buffer overnight at 4 ℃ and washed by PBS for three times after incubation. The vesicles were injected into melanoma tumor bearing C57BL/6 mice through tail-vein. After 24 h, the tumors and major organs were harvested. The fluorescence imaging and intensities were detected with Xenogen IVIS Spectrum imaging system.

### *In Vivo* Bioluminescence Imaging

The luciferase-tagged B16F10 melanoma tumor cells were injected under the left dorsal skin of C57BL/6J mice. On the sixth day, the mice were divided into six groups. Mice were intravenously injected with PBS, CD64-NVs, CD64-NVs-aPD-L1, CD64-NVs-CP, aPD-L1+CP and CD64-NVs-aPD-L1-CP. The tumor burden was detected via bioluminescence after L-luciferin injection with an IVIS Lumina imaging system. Animal experiments were performed under an approved protocol by the Administrative Committee of Animal Research in the Sun Yat-Sen University.

### Tissue Immunofluorescence Assay

The tumors were removed from anesthetized animals and instantly frozen in optimal cutting medium (O.C.T.). Ten micrometer sections were cut with a cryotome and mounted on slides. The tumor sections were soaked in PBS for 15 min and blocked with the buffer including 3% BSA for 30 min. And then, the sections were incubated with CD4 and CD8 primary antibodies overnight and washed with PBS. The sections then were incubated with Alex Fluor 647 and Alex Fluor 488 secondary antibodies at room temperature in the dark for 1 h. Finally, the nucleus was stained with DAPI and washed with PBS. Confocal microscopy was performed on a FLUO-VIEW laser scanning confocal microscope (Zeiss) in sequential scanning mode using a 40× objective.

### Statistical Analysis

All results are expressed as mean ± SD or the mean ± SEM as indicated. Biological replicates were used in all experiments unless otherwise stated. Statistical graph software Spss16.0 was used to calculate the difference of data in different groups. Differences between groups were compared by One-way or two-way analysis of variance (ANOVA) and Tukey post-hoc tests as indicated. *P*<0.001 was considered as extremely significant difference, *P*<0.01 as significant difference, and *P*<0.05 as statistical difference.

## Results and conclusion

### Schematic of the production and characterization of CD64 presenting NVs

**Figure 1:**
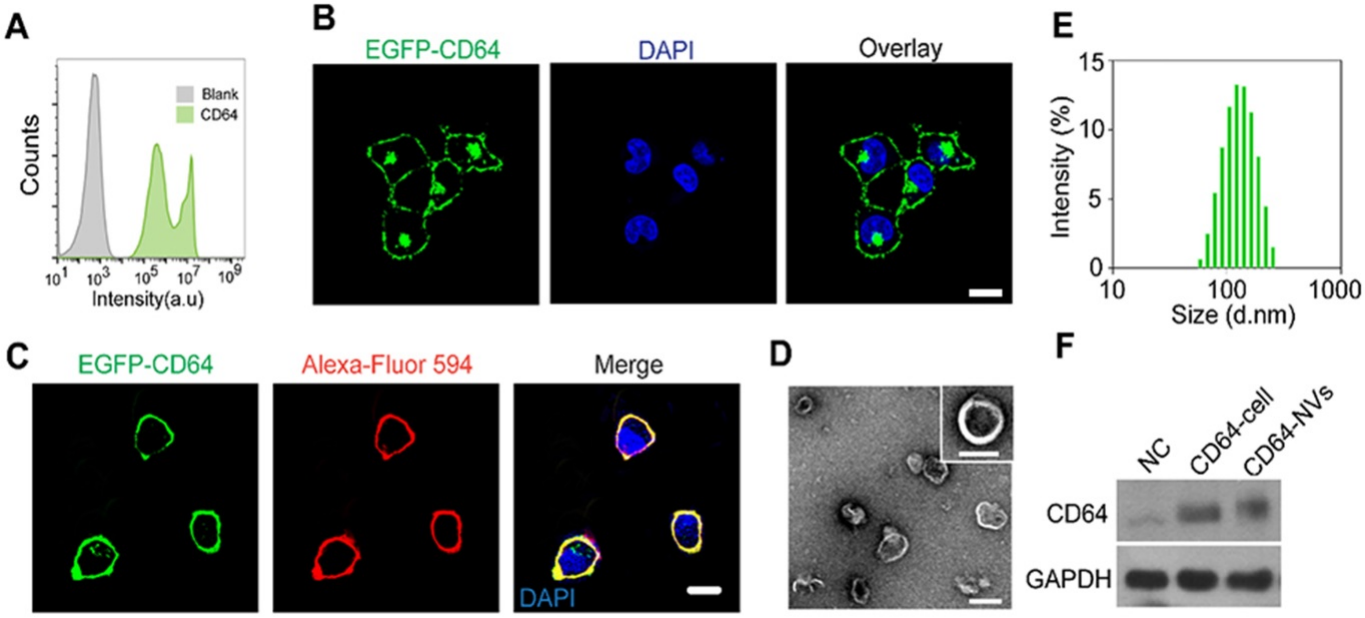
Characterization of CD64 presenting NVs. (A) Analysis of EGFP-CD64 expression of HEK 293T cells after transfection without screening by flow cytometry. (B) Establishment of HEK 293T cell line steadily expressing CD64 after screening. Scale bar: 10 µm. (C) The confocal image of the HEK 293T cell line stably expressing mouse EGFP-CD64 on the cell membrane. WGA Alexa-Fluor 594 dye was used to stain cell membrane. Scale bar: 10 µm. (D) The TEM image of CD64-NVs suggesting the shape and size. Scale bar: 100 nm. (E) The size distribution of CD64-NVs measured by dynamic light scattering (DLS). (F) The western blot analysis indicated the expression of CD64 of HEK 293T cells and NVs. NC: HEK 293T cells without transfection, as negative control. GAPDH was used as loading control.

In order to prepare CD64-NVs, we established the HEK 293T cell lines stably expressing CD64 on the cell membrane. HEK 293T cell is often considered as a engineered cell line because it can be easily utilized to express exogenous recombinant proteins and retroviruses after being transfected with plasmids in the biology research and industry 50-52. GFP-Spark protein-tag was designed to attach on the C-terminal of CD64 protein, located in the intracellular membrane, while the interaction domain with Fc of CD64 was on the outside of the membrane (Scheme 1). Therefore, we cloned the mouse CD64 cDNA to pCMV3, a mammalian expression vector. The transfected HEK 293T cells were observed under the light microscope without selecting. We found that the majority of HEK 293T cells emerged green fluorescence which indicated the successful transfection. Furthermore, flow cytometry analysis showed that the percentage of green fluorescence positive cells was more than 90% (Figure 1A). To further obtain stable transfected cell lines, we screened the stable cell lines with hygromycin B. We further isolated the monoclonal cell from CD64 cell line to perform monoclonal amplification in order to obtain optimal cell line with intensive expression of CD64 protein (Figure 1B). Remarkably, CD64 was localized on the membrane as indicated by the localization of the EGFP fluorescence and the red fluorescence of Alexa-Fluor 594 conjugated wheat germ agglutinin (WGA) on the membrane (Figure 1C).

Next, the optimal CD64 cell line was largely cultured in order to collect the cell membrane. The cell membrane vesicles were prepared by extruding through 0.8 and 0.22 µm pore-sized hydrophilic membrane filters. The morphology of the CD64-NVs was detected by the transmission electron microscopy (TEM) (Figure 1D). The distribution of CD64-NVs was detected by the dynamic light scattering (DLS), which showed that the particle size was about 100 nm with the average zeta potential of -12 mV (Figure 1E and Figure S1). Coomassie blue staining showed that there was no significant difference between CD64-NVs and CD64 cell lines in the amount of CD64 protein, indicating that there was a small loss of cell membrane during the preparation of CD64-NVs (Figure S2). The expression of CD64 protein on the NVs was further verified by western blot (Figure 1F).

### *In vitro* biological behavior and *in vivo* biodistribution of CD64-NVs

**Figure 2:**
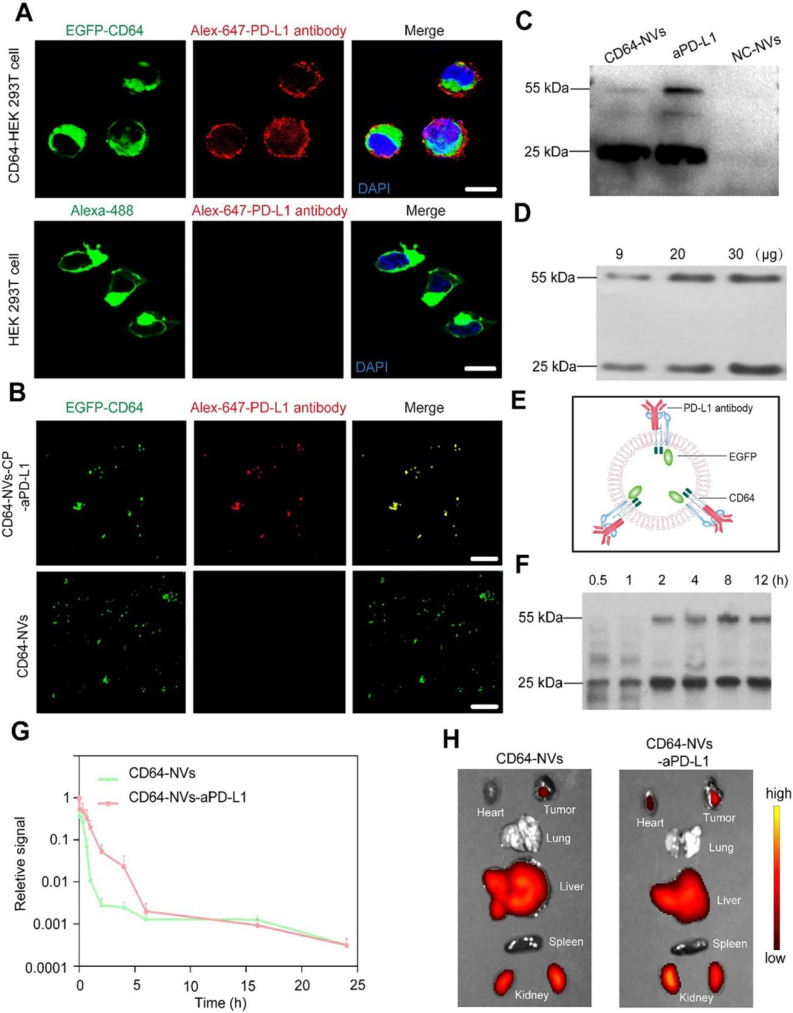
*In vitro* biological behavior and *in vivo* biodistribution of CD64-NVs. (A) HEK 293T cells stably expressing EGFP-CD64 bound the Fc of PD-L1 antibodies on the membrane. The cells were incubated with PD-L1 antibodies for 4h. WGA Alexa-Fluor 488 dye and 647 dye were used to detect HEK 293T cell membrane and PD-L1 antibodies, respectively. Scar bar: 10 µm. (B) Immunofluorescence image indicated the co-localization of CD64-NVs with PD-L1 antibodies. Scar bar: 5 µm. (C) Western blot analysis indicated the interaction between CD64 (on NVs) and PD-L1 antibodies. 25 kDa represented the light chain of PD-L1 antibody. aPD-L1 acted as the positive control. NC-NVs (non-transfection cell membrane nanovesicles) worked as the negative control. (D) Different dosages of CD64-NVs were incubated with 2 μg PD-L1 antibodies for 4 h and detected by western blot. (E) Schematic of CD64 on the NVs acted as a linker to catch PD-L1. (F) Western blot analysis was used to determine the release of PD-L1 antibodies from CD64-NVs at different time points as indicated. (G) Cy5.5 labeled CD64-NVs and CD64-NVs-aPD-L1 were injected into mice by tail intravenous injection. Fluorescence signal intensity was measured at different time points as indicated. (H) The IVIS spectrum image showed the distribution of CD64-NVs and CD64-NVs+aPD-L1 in tumor and major organs, including heart, lung, liver, spleen and kidney.

The multiple types of tumor cells express PD-L1 to exhaust cytotoxic CD8^+^ T cell by interacting with PD-1 receptor 40, 53. To investigate whether CD64 could capture PD-L1 antibodies, we incubated CD64 presenting cells with PD-L1 antibodies for 4 h *in vitro*. We observed that the green fluorescence of GFP-CD64 on the cell membrane was overlapped by the red fluorescence of PD-L1 antibodies, which demonstrated that CD64 proteins could positively capture PD-L1 antibodies. In contrast, CD64-free cells had limited ability to combine with PD-L1 antibodies (Figure 2A). Furthermore, the colocalization of the green and red fluorescence also confirmed the binding between CD64-NVs and PD-L1 antibodies (Figure 2B). To further investigate the antibody capture ability of the CD64-NVs, we detected the heavy chains (H chain) and the light chain (L chain) of PD-L1 antibodies by western blot. Virtually, CD64-NVs could effectively capture PD-L1 antibodies (Figure 2C). To determine the optimal dosage of CD64-NVs to combine with PD-L1 antibodies, we incubated the different dosages of CD64-NVs with 2 μg PD-L1 antibodies for 4h. The dose of CD64-NVs at 30 μg has the optimal combination effect (Figure 2D and Figure S3). CD64 acted as a linker to catch PD-L1 antibodies on the surface of NVs (Figure 2E). To explore the release of PD-L1 antibodies and CP *in vivo*, we measured the release amount through simulation environment. The PD-L1 antibodies were released from CD64 NVs within 2 h. Afterwards, the release of PD-L1 antibodies became slow (Figure 2F). Next, to detect the loading and releasing ratio of CP, we loaded CP into NVs and measured the loading efficiency and release speed *in vitro* (Figure S4A-C). As shown in Figure S4, CP could be released from NVs into the solution dependent a time course. Meanwhile, loading of CP do not affect the properties of CD64-NVs (Figure S4B and Figure S5).

To investigate the biodistribution in internal organs and pharmacokinetic characteristics of CD64-NVs, we labeled CD64-NVs and CD64-NVs-aPD-L1 with Cy-5.5 dye. The NVs were injected into mice through intravenous injection. The curve showed that the fluorescence intensity in the blood of mice in the CD64-NVs-aPD-L1 group decreased slowly before 6 h compared with the CD64-NVs group. Although the changes of fluorescence intensity in the two groups were not significant, but to some extent, CD64-NVs-aPD-L1 remained in mice for longer time (Figure 2G). Next, for biodistribution in the internal organs of NVs, the mice were treated with CD64-NVs and CD64-NVs-aPD-L1 by tail vein injection. We observed the accumulation of the NVs in liver, spleen, lung, kidney and tumor. The fluorescence intensity of tumor was a bit higher in the CD64-NVs-aPD-L1 group, compared with the CD64-NVs group (Figure 2H and Figure S6).

### *In vivo* anti-tumor effect of CD64-NVs-aPD-L1-CP

**Figure 3:**
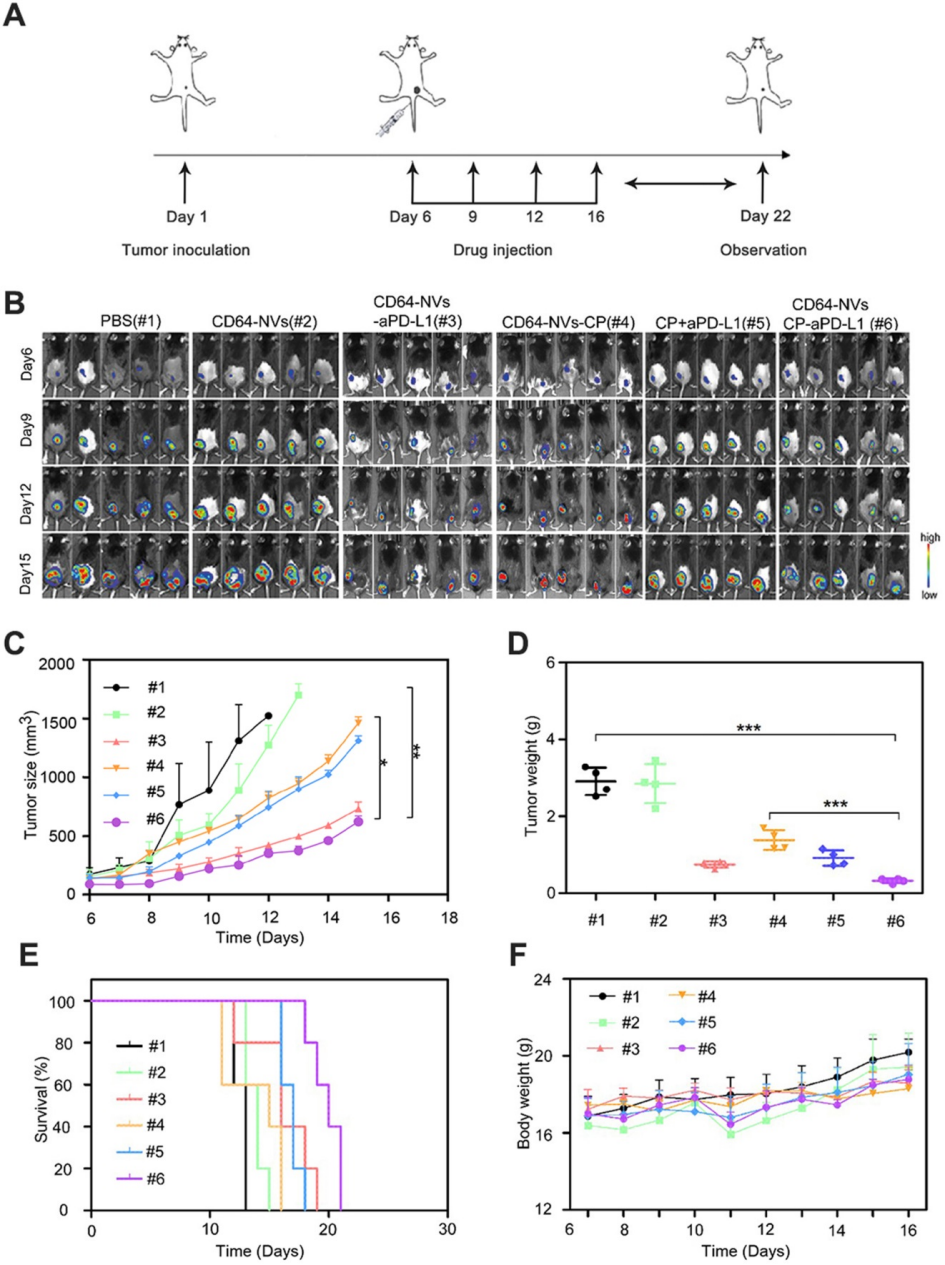
*In vivo* anti-tumor effect of CD64-NVs-aPD-L1-CP. (A) Schematic illustration of CD64-NVs-aPD-L1-CP used for therapy in the B16F10 melanoma model. (B) *In vivo* bioluminescence imaging of the B16F10 melanoma tumor growth of different mice treated with PBS (#1), CD64-NVs (#2), CD64-NVs-aPD-L1 (#3), CD64-NVs-CP (#4), CP+aPD-L1 (#5) and CD64-NVs-CP -aPD-L1 (#6) at different time points (*n*=5). (C) Average tumor sizes for the treated mice (*n*=5). The experimental data were shown as mean ± SEM. (D) Tumor weights isolated from euthanized mice in each group (*n=*4). (E) Survival curves for the mice treated with PBS, CD64-NVs, CD64-NVs-aPD-L1, CD64-NVs-CP, CP+aPD-L1 and CD64-NVs-CP -aPD-L1 (*n=*5). (F) Body weights of mice receiving the different treatment and control mice (*n=*5). Mean ± SEM, error bar. CD64-aPD-L1 and CP were considered as two factors. The second two-way ANOVA with Tukey post-hoc test was carried out between PBS (#1), CD64-NVs (#2), CD64-NVs-aPD-L1 (#3), CD64-NVs-CP (#4), CP+aPD-L1 (#5) and CD64-NVs-CP-aPD-L1 (#6) groups. Throughout, *P < 0.05, **P < 0.01, ***P < 0.001; by one-way analysis of variance (ANOVA) with C, D. Tukey post-hoc tests or by Log-Rank (Mantel-Cox) test.

To investigate the synergistic antitumor effect of CD64-NVs-CP-aPD-L1, we established the mouse melanoma model to evaluate the efficacy by hypodermic injection of B16F10-luci cells in C57BL/6 mice. On the sixth day of tumor inoculation, the mice were randomly divided into six groups, including PBS group (Group 1), CD64-NVs group (Group 2), CD64-NVs-aPD-L1 (Group 3), CD64-NVs-CP (Group 4), aPD-L1+CP group (Group 5) and CD64-NVs-CP-aPD-L1 group (Group 6), and drug treatments were started later (Figure 3A). We evaluated the anti-tumor effect of different treatments by detecting bioluminescence signal intensity and measuring tumor sizes. Distinctly, CD64-NVs-CP-aPD-L1 could intensively suppress the melanoma tumor growth (Figure 3B-D). Meanwhile the mice received CD64-NVs-CP or aPD-L1 plus CP group also delay the tumor growth (Figure 3B-D). Nevertheless, CD64-NVs exhibit excellent antibody and drug-controlled release, which lead the benifit of the treatment. Moreover, CD64-NVs-CP-aPD-L1 group improved the survival time of mice without significant change in body weight (Figure 3E-F). We also collected major organs to assess the systemic toxicity including heart, liver, spleen, lung and kidney. There was no obvious damage to organs in the CD64-NVs-CP -aPD-L1 group (Figure S7).

### CD64-NVs-aPD-L1-CP enhanced anti-tumor effect *in vivo* by promoting CD8^+^ T cell proliferation and inhibiting Tregs activity

**Figure 4:**
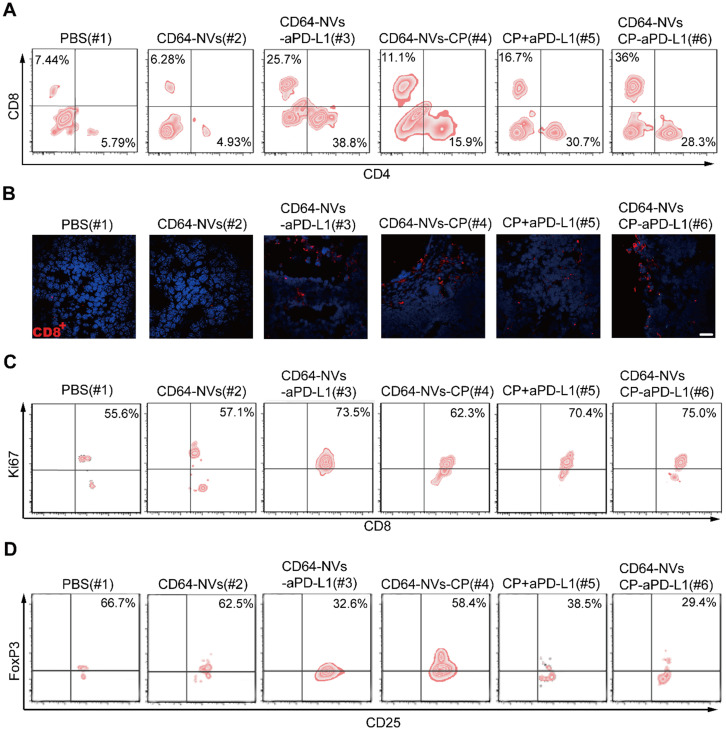
CD64-NVs-CP-aPD-L1 enhanced anti-tumor effect *in vivo* by promoting CD8^+^ T cell proliferation and inhibiting Tregs activity*.* (A) Representative plots of T cells in tumors of different treatment detected by flow cytometry (Gated on CD3^+^). (B) Representative image of immunofluorescence staining of the tumor sections showed CD8^+^ T cells infiltration (Scar bar: 20 µm). (C) Representative plots of Ki67 in CD8^+^ T cells infiltrating in tumors detected by the flow cytometry (gated on CD3^+^). (D) Representative plots of Foxp3 in Tregs infiltrating in tumors detected by the flow cytometry (gated on CD4^+^).

Previous studies have shown that tumor microenvironment is mainly an immunosuppressive state where there are complex components, such as cancer cells, immune cells and cytokines 54-56. PD-L1 antibody can relieve the inhibition of CD8^+^ T cells and CD4^+^ T cells caused by PD-L1 positive tumor cells 57. Previous study has demonstrated that low dose of CP could inhibit the activity of Tregs and activate CD8^+^ T cells 54. To assess whether CD64-NVs-CP-aPD-L1 formulation further activates CD8^+^ T cell to exert antitumor activity, we investigated the frequencies of CD8^+^ T cells infiltrating within the tumor detected by flow cytometry. We found that the percentage of CD8^+^ T cells was significantly increased in the tumor of mice received the treatment of CD64-NVs-CP-aPD-L1 (Figure 4A). Similarly, CD64-NVs-CP-aPD-L1 treatment enhance the CD8^+^ T cells infiltrated into the tumor, which were detected by immunofluorescent cytochemical staining (Figure 4B). There exhibit more infiltrated T cells in tumor compared with the mice treated with CD64-NVs-CP or aPD-L1 plus CP group (Figure 4B), which directly attribute the elimination of cancer cells. Remarkably, the percentage of Ki67^+^ T cells was significantly increased in CD64-NVs-CP-aPD-L1 treated mice, which implied that it could promote CD8^+^ T cell proliferation (Figure 4C). Meanwhile, CD64-NVs-CP-aPD-L1 had the effect in reducing CD4^+^ FoxP3^+^ T cells, which could relieve the inhibition of CD8^+^ T cells and further activate CD8^+^ T cell (Figure 4D). Altogether, these results revealed that CD64-NVs-CP-aPD-L1 could simultaneously interrupt the immunosuppressive effect of PD-L1 and decrease the inhibition of Tregs, contribute to tumor suppression.

## Conclusion

In summary, we engineered cellular NVs displaying CD64, which could capture antibodies by binding to the Fc domain without affecting their bioactivity. Virtually, PD-L1 on the melanoma tumor cells was blocked by CD64-NVs-aPD-L1 formulation *in vivo*, which promoted the anti-tumor effect of CD8^+^ T cells by enhancing activation and proliferation. Moreover, CD64-NVs-aPD-L1 could be considered as promising drug carrier to achieve the possibility of combination therapy. CP, an inhibitor of Tregs, was loaded into CD64-NVs-aPD-L1 and intravenously injected into melanoma mice. Hence, CD64-NVs-CP-aPD-L1 formulation liberated the effect T cells from PD-L1 and Tregs. The double promotion of T cell activation could inhibit tumor growth, improve the quality of life and extend survival time of the tumor-bearing mice. Thus, CD64-NVs are charismatic drug carriers that could achieve both checkpoint blockade antibody and immunomodulatory chemical drugs for combined cancer immunotherapy.

## Supplementary Material

Supplementary figures.

## Abbreviations

NVs: nanovesicles
PD-1: programmed cell death protein 1
PD-L1: Programmed cell death ligand 1
CTLA-4: cytotoxic T lymphocyte-associated antigen-4
Tregs: Regulatory T cells
CP: cyclophosphamide
IL-2: interleukin-2
Fc: crystalline fragment
IgG: Immunoglobulin G
DMEM: Dulbecco's modified Eagle's medium
FBS: fetal bovine serum
HEK 293T: Human Embryonic Kidney 293T
HM: homogenization medium
NHS-Cy5.5: Cyanine5.5 NHS ester
WGA: germ agglutinin
TEM: transmission electron microscopy
DLS: dynamic light scattering
