# Supplementary material for: Genetic engineering cellular vesicles expressing CD64 as checkpoint antibody carrier for cancer immunotherapy
Source: Theranostics. 2021 Apr 7;11(12):6033–43. doi: 10.7150/thno.48868 (PMC8058713; doi:10.7150/thno.48868)
Supplement: Supplementary file 1 — Supplementary figures. [file thnov11p6033s1.pdf]

Supporting Information

Genetic engineering cellular vesicles expressing CD64 as checkpoint antibody carrier for cancer immunotherapy

Liyan Li[a#], Qianwei Miao[d#], Fanqiang Meng[a#], Baoqi Li[a], Tianyuan Xue[a], Tianliang Fang[a], Zhirang Zhang[a], Jinxie Zhang[c], Xinyu Ye[d], Yang Kang[e], Xingding Zhang[a], Qian Chen[f], Xin Liang[b, g*], Hongbo Chen[c*], Xudong Zhang[a*]

a. Department of Pharmacology, Molecular Cancer Research Center, School of Medicine, Sun Yat-Sen University, Guangzhou/Shenzhen, China

b. Guangdong Provincial Key Laboratory of Medical Molecular Diagnostics, Key Laboratory of Stem Cell and Regenerative Tissue Engineering, School of Basic Medical Sciences, Guangdong Medical University, Dongguan 523808, China.

c. School of Pharmaceutical Sciences (Shenzhen), Sun Yat-Sen University, Shenzhen, P.R. China.

d. School of Life Sciences, Tsinghua University, Beijing 100084, P.R. China.

e. The Seventh Affiliated Hospital, Sun Yat-sen University, Shenzhen, 518107, China.

f. Institute of Functional Nano & Soft Materials (FUNSOM), Soochow University, Suzhou, 215325, P.R. China.

g. Center for Experimental Medicine (CEM), University of Chinese Academy of Sciences-Shenzhen Hospital, Shenzhen 518000, P. R. China

#These authors contributed equally to this work.

*Corresponding Author: E-mail: zhangxd56@mail.sysu.edu.cn

E-mail: chenhb7@mail.sysu.edu.cn

E-mail: liangx55@mail.sysu.edu.cn

Supplementary Figures

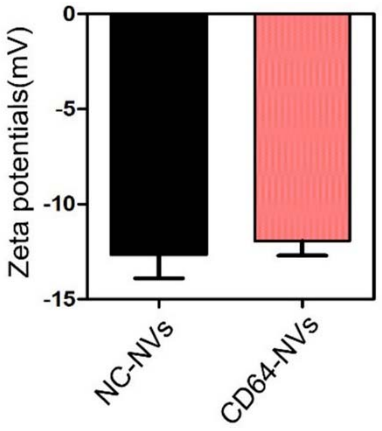

Figure S1. The zeta potential of the CD64-NVs. NC: non-transfecting control, worked as a negative control.

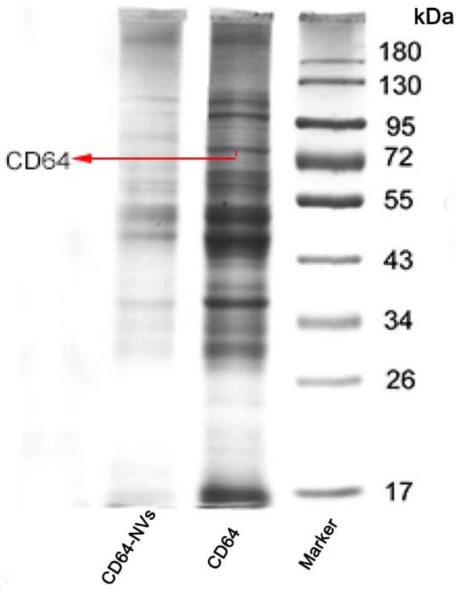

Figure S2. The expression of CD64 on the NVs. The image of staining with coomassie brilliant blue showed that the existence of CD64 on the NVs.

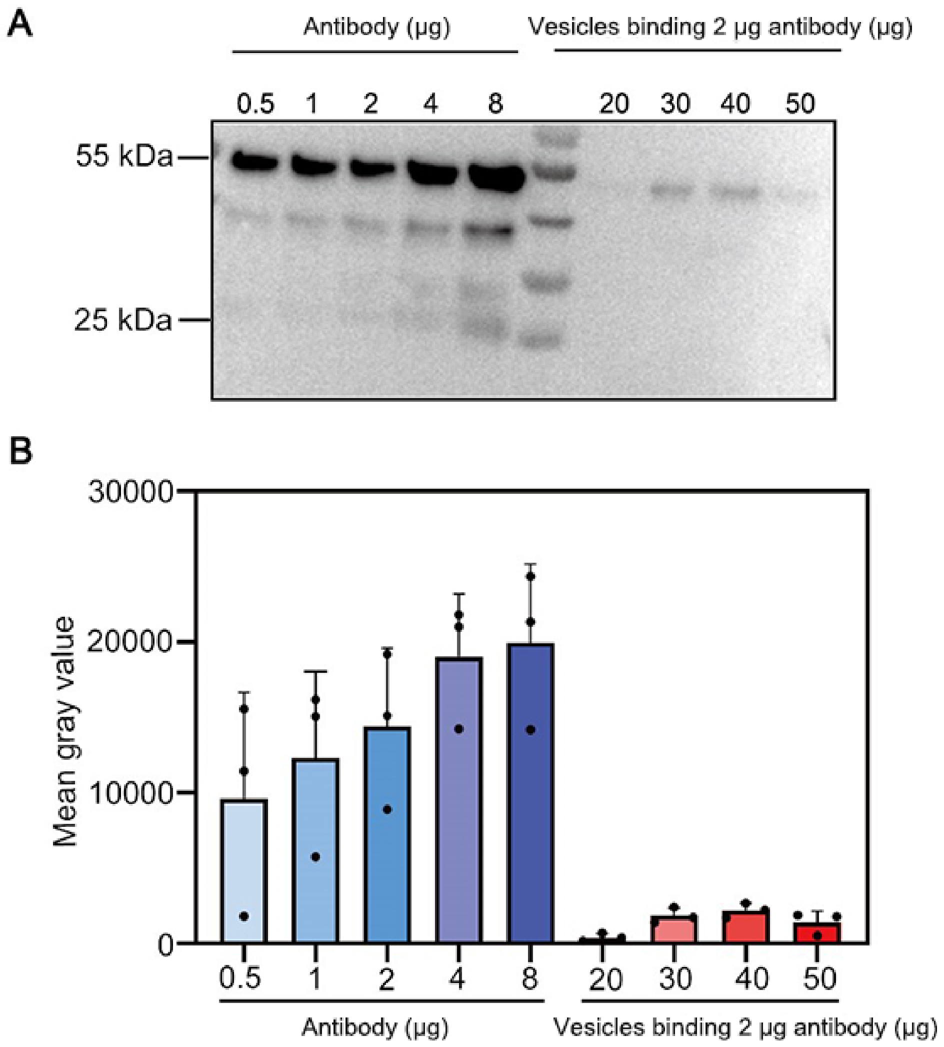

Figure S3. (A) Western blot analysis was used to examine different dosages of CD64-NVs binding to 2 μg PD-L1 antibody. 0.5, 1, 2, 4, 8 μg PD-L1 antibodies were used as standard control. (B) Quantitative analysis of band intensity by WB (*n*=3). Error bar, mean ± s.d.#

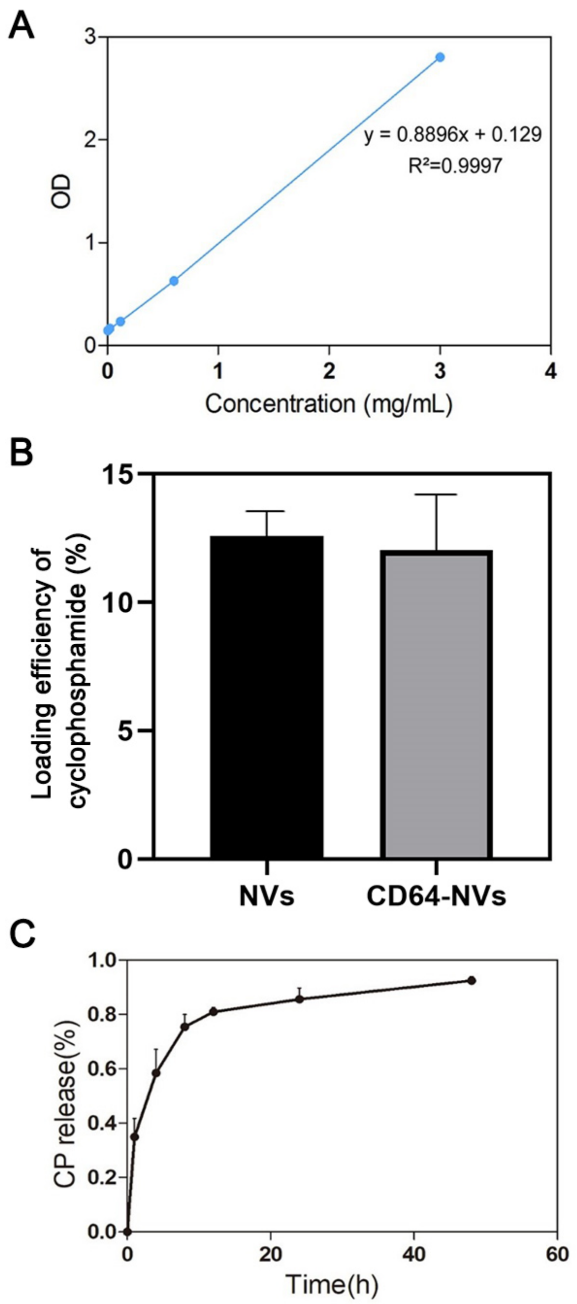

Figure S4. CP released from CD64-NVs-CP at different time points. (A) The Standard curve of CP. (B) CP loading in NVs and CD64-NVs (n=3). Error bar, mean ± s.d. (C) The release of CP at different time points.

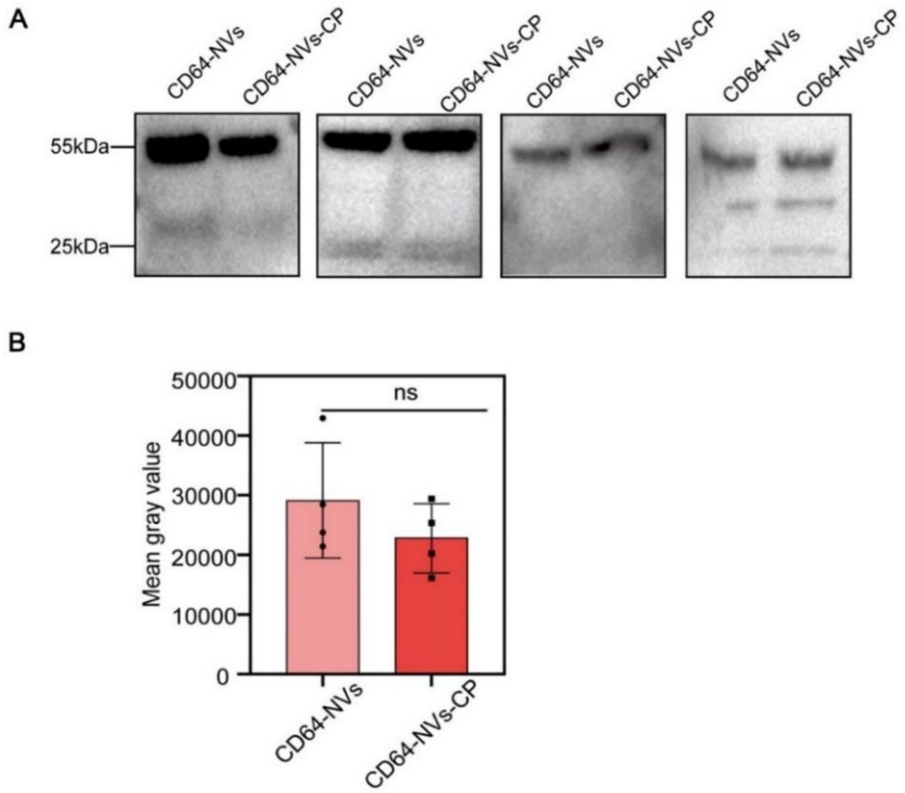

Figure S5. (A) Western blot was used to detect the effect of the loading of CP on the binding ability of CD64-NVs to PD-L1 antibody (n=4). 55 kDa represented the heavy chain of PD-L1 antibody, 25 kDa represented the light chain of PD-L1 antibody. (B) Mean gray value analysis of band intensity by WB (n=4).

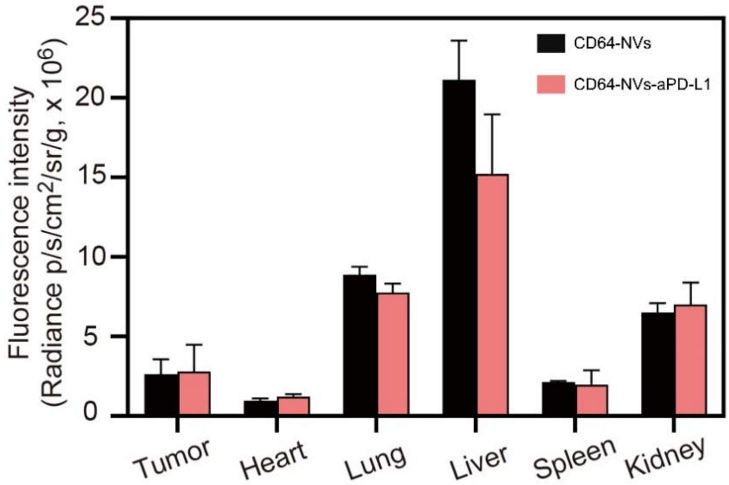

Figure S6. Fluorescence intensity per gram tissue in tumor and major organs measured (n=3). Error bar, mean ± s.d.

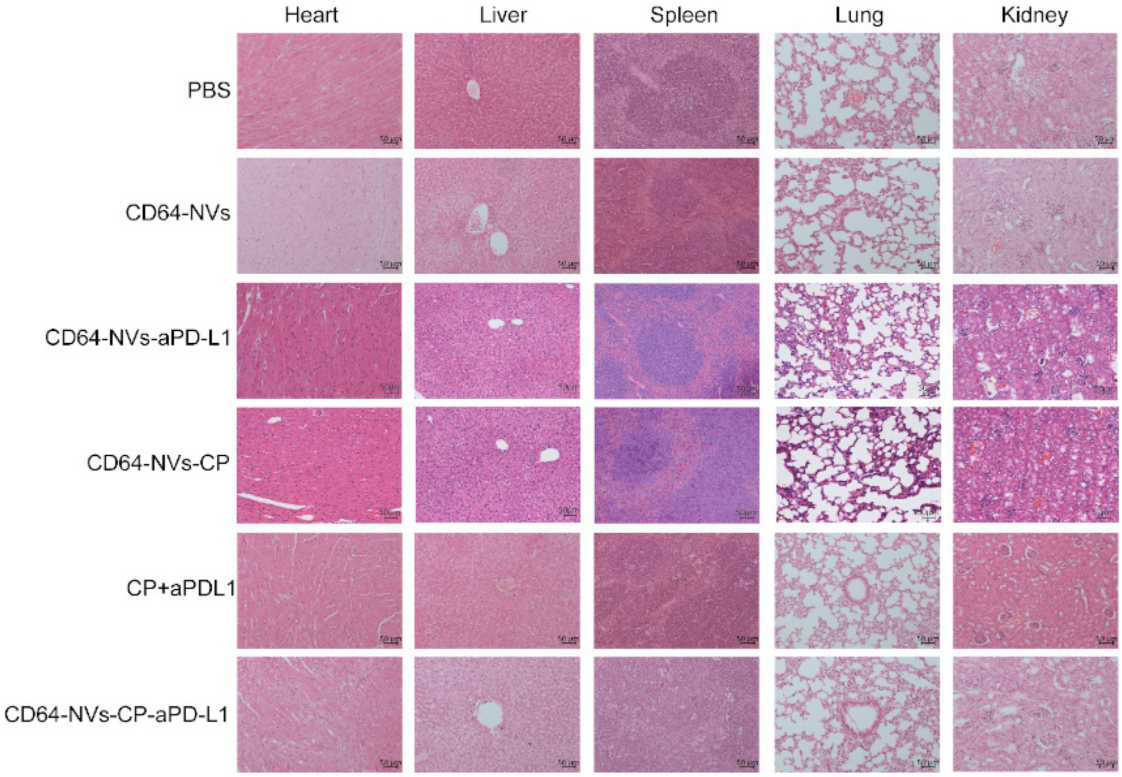

Figure S7. The image of H&E staining obtained from heart, liver, spleen, lung and kidney of mice treated with PBS, CD64-NVs, CD64-NVs-aPD-L1, CD64-NVs-CP, CP+aPD-L1 and CD64-NVs-CP-aPD-L1, respectively. Scale bars: 50 μm.
